# The role of photobehaviour in sponge larval dispersal and settlement

**DOI:** 10.1371/journal.pone.0287989

**Published:** 2023-07-10

**Authors:** Steve Whalan

**Affiliations:** Faculty of Science and Engineering, Southern Cross University, Lismore, New South Wales, Australia; King Abdulaziz University, SAUDI ARABIA

## Abstract

Deciphering the behavioural ecology of adult (sessile) sponges is challenging. However, their motile larval stages afford opportunities to investigate how behaviour contributes to dispersal and selection of habitat. Light is a fundamental cue contributing to larval sponge dispersal where photoreceptive cells contribute to this process. But how universal is light as a cue to sponge larval dispersal and settlement? Behavioural choice experiments were used to test the effect of light on dispersal and settlement behaviours. Larvae of the tropical sponge species *Coscinoderma mathewsi*, *Luffariella variabilis*, *Ircinia microconnulosa*, and *Haliclona* sp., from deep (12–15 m) and shallower-water habitats (2–5 m), were used in experiments. Dispersal experiments provided a light-gradient-choice where light represented light attenuation with depth. Light treatments included white light and the spectral components of red and blue light. Settlement experiments comprised a choice between illuminated and shaded treatments. Fluorescence microscopy was used to establish the presence of fluorescent proteins associated with posterior locomotory cilia. Deeper-water species, *C*. *mathewsi* and *I*. *microconnulosa* discriminate light spectral signatures. Both species changed dispersal behaviour to light spectra as larvae aged. For *C*. *mathewsi* positive phototaxis to blue light changed to photophobic responses (all light treatments) after six hours and behaviours in *I*. *microconnulosa* changed from positive to negative phototaxis (white light) after six hours. *L*. *variabilis*, also a deeper-water species, was negatively phototactic to all light treatments. Larvae from the shallow-water species, *Haliclona* sp., moved towards all light wavelengths tested. There was no effect of light on settlement of the shallow-water *Haliclona* sp., but larvae in all three deeper-water species showed significantly higher settlement in shaded treatments. Fluorescence microscopy showed discrete fluorescent bands contiguous to posterior tufted cilia in all four species. These fluorescent bands may play a contributory role in larval photobehaviour.

## Introduction

Animals with complex life cycles are ideal model organisms to investigate questions on ecology and evolutionary biology [1]. Complex life cycles can include organisms with a single-generation life history defined by distinct ontogenetic changes established through metamorphosis [1]. Metamorphosis, in its broadest definition, is a transformation process across life cycle groups typically exemplified through a transition from a larval to juvenile or adult form [2]. While the transition from larvae to juvenile commonly coincides with notable changes in morphology, physiology and behaviour it can also include a change in habitat (environmental conditions) [3, 4], thereby highlighting the capacity for the sequential occupation of two niches [2, 5]. Moreover, larval phases exposed to different habitats, experiences, or environmental conditions, can contribute to adult traits and performance and, therefore, whole-life fitness, particularly for transitions of motile larval into sessile adults [6–10].

There are well-defined examples of the linkage between larval stages and resultant adult performance and persistence in sessile marine invertebrates [11]. For this group, the motile larval phase contributes to species (adult) habitat distributions. Successful recruitment to adult populations relies on larval dispersal and settlement to suitable/optimal habitats, often in response to specific settlement cues. Environmental-based cues contributing to settlement in sessile marine invertebrate groups can be chemical-based [12, 13] or physical-based [14–16]. Light, associated with depth or turbidity, is a physical cue that can influence larval phototaxis, thereby directing dispersal and settlement and, more broadly, distributions of marine invertebrates [17–21].

Organism phototactic behaviour can involve attraction or avoidance to light gradients and are important to a broad spectrum of life, spanning both prokaryotes and eukaryotes [22–24]. For complex life cycle animals, larval phototaxis can enable planktonic dispersal through vertical migration within the water column, thereby contributing to population demographics and connectivity [25]; larval phototactic behaviours of sessile invertebrates can also influence settlement choices [26] with implications for adult performance and fitness [11]. This is particularly noted for autotrophic marine sessile invertebrates (e.g. corals) where adult performance is established in habitat that accesses adequate sunlight to photosynthesize but at depths where light attenuates to limit light (UV) stress [27, 28].

Effective phototaxis in sessile marine invertebrate larvae relies on a biological sensory system or photoreceptors to perceive and respond to light sources (e.g. celestial bodies of the moon or sun). A diverse range of photoreceptors are found in single and multicellular pelagic organisms but are universally underpinned by a fixed, polarized body shape that exhibits spiral swimming propelled and orientated by cilia coordinated by photosensory systems [22]. A review by Thorson [21] highlighted phototactic behaviours in a wide range of marine invertebrate larvae to visible light. More recent work has identified the capacity of larvae to respond to different wavelengths of light [29–32] with an increasing focus on sensory systems utilized to respond to light [30, 33, 34].

The form and function of visual sensory systems within marine invertebrate larvae are diverse, ranging from photoceptor/pigmented type cells to structured eyes [35]. An underlying feature of most animal photosensory systems is the coupling to a neural platform [35]. Neural machinery is observed in most metazoans but for basal groups exhibiting phototactic behaviour [30, 31, 36–40], nervous systems are rudimentary (e.g. cnidarians) or lacking (i.e. sponges), and for sponges, in particular, raise interesting questions around the mechanisms of behavioural responses to light intensity and wavelengths. How these basal invertebrates sense and respond to light has been centred on the use of photoreactive opsins and photolyase/cryptochrome proteins [30]. Are there other photosensory systems that contribute to photobiology in marine taxa? The role of fluorescent proteins contributing to light-moderating processes in fishes has highlighted a functional role in intra-specific signalling [41, 42]. Fluorescent proteins are also reported in cnidarians, with proposals that these proteins contribute to photoprotection [43, 44] and in light sensing [45–47]. Other examples do not support the role of fluorescent proteins in light detection [48], so the question of these proteins contributing to light sensing in cnidarians is still to be fully resolved. Any role fluorescent proteins may play in sponge larval photobiology has not been established.

Many sponge larvae have a pigmented cellular band adjacent to a posterior ciliated tuft that propels and directs a larva. Seminal work by Leys and Degnan [38] and Leys et al. [37] linked the phototactic response in larvae of *Amphimedon queenslandica* to the pigmented cellular ring; these authors suggested flavins, carotenoids or cryptochromes may be important to larval light detection. Further molecular work by Rivera et al. [34] confirmed the work of Leys et al. [37] reporting on cryptochrome-based genes in this sponge with associated proteins expressed at the pigmented ring region having a flavin-based cofactor that reacts to light.

While evidence builds for larval phototactic behaviours in several species of sponge, finer details on whether sponge larvae can discriminate spectral components of light to aid dispersal represents a knowledge gap. To begin to meet this gap, this study aimed to establish if sponge larvae detect, and respond to, light that attenuates with depth, thereby influencing dispersal, settlement and depth distributions of adults. Species from shallow and deeper water habitats were used in (1): dispersal experiments exposed to light wavelengths representing light attenuation with depth, including white, red and blue light and (2): settlement assays to light and shaded treatments. A secondary focus included a qualitative measure of the presence of fluorescent proteins contiguous to posterior cilia in larvae from all four species to aid interpretations of this trait contributing to dispersal and settlement photobehaviours.

## Materials & methods

### Sponge larval collection

Four species of sponge were targeted: *Coscinoderma mathewsi*, *Luffariella variabilis*, *Haliclona* sp. and *Ircinia microconnulosa*. These species are gonochoristic, viviparous, and dribble spawn larvae diurnally during the Austral summer [49–51]. Sponges were collected from inshore fringing reefs of Orpheus and Fantome Islands, which form part of the Palm Island group on the central Great Barrier Reef (GBR). Between five and 13 adult sponges from each species were collected and transported to Orpheus Island Research Station, where they were maintained in flow-through aquaria. Larvae were collected using traps placed over sponges, following established methods to collect brooded sponge larvae [40]. Collections and research were carried out under Marine Parks permit, G21/44978.1, issued by the Great Barrier Reef Management Authority.

### Depth distributions

All four species of sponge occur throughout the Palm Islands. To establish depth distributions of each species, surveys were conducted at five sites: Little Pioneer Bay (18° 35.5831 S 146° 29.0113 E), Cattle Bay (18° 36.8813 S 146° 28.9888 E), Hazard Bay (18° 35.2545 S 146° 30.9638 E), Juno Bay (18° 39.5922 S 146° 29.0113 E), and Pelorus Island (18° 32.7594 S 146° 29.2577 E). Surveys were conducted at depth profiles of ~2–5 m, 6–9 m and 10–12 m, recording each target sponge species encountered along with its depth. Each survey was approximately 40 mins. The effect of depth on species distributions was analysed with Welch’s ANOVA and Tukey’s post hoc test.

### Dispersal

A light-choice chamber was used to determine larval dispersal responses to light (S1 Fig). The chamber comprised two 176 mm high pipes (110 mm diameter PVC) which were orientated vertically and capped at the bottom to hold water. Both vertical pipes were plumbed into a central 540 mm long horizontal connector pipe (35-mm diameter PVC) so that water would fill both vertical and horizontal sections. One of the vertical pipes had a 20 mm hole drilled into it, which was subsequently sealed with a clear acrylic disc (30 mm diameter x 3 mm thick) so that an external light source (Ledlenser—T2QC Quad-Colour Flashlight) could penetrate through the acrylic window of the vertical upright and then along the 35 mm connector pipe to provide a light gradient across the horizontal section of the chamber. Specifically, the immediate entry point of the light source established a maximal intensity of light which then diffused through the connector section and terminated at the opposite vertical upright which provided a minimum intensity of light. The objective was to provide a choice between intense light from three different wavelengths (white 400–720 nm, red 650–700 nm and blue 430–470 nm) at one end of the chamber and less intense light at the opposing end so larval responses of attraction to, or avoidance of, the light source could be measured. The connector pipe had a section removed at the top (360 mm) to provide an opening that allowed larvae to be introduced to the “choice-chamber” and that could be viewed from above to record larval directional responses. The entire choice-chamber was filled with 2 litres of FSW (20 μm) and the light source, held in place with a retort stand, was orientated into the clear acrylic window.

Once the choice-chamber with the light source was established a single larva was carefully pipetted into the central section of the connector pipe and monitored for up to two minutes, noting the swimming direction of larvae within the connector pipe. The centre included an arbitrary buffer zone of five cm from the central insertion point. Larval responses to each light treatment were noted as attraction or avoidance, or no movement from the centre. A successful response (for either attraction or avoidance) relied on each larva moving from the centre to > 50% of the distance, in either direction, within the connector pipe. To reduce any experimental bias of the choice-chamber orientation, two choice-chambers were used interchangeably, and each trial consisted of orienting the choice-chamber, and light source in different positions. Each larval trial was considered a replicate. Experiments were conducted in a darkened laboratory where the temperature was maintained at 28°C (ambient sea water temperature). To determine if dispersal behaviour changed as larvae aged, an initial experimental phase used larvae that were 0.5–1 h old, hereafter termed early, and a separate phase used larvae that were 4–6 h old, hereafter termed late, (see S1 Table for sample sizes).

The effect of white, red and blue light on the movement patterns of larvae was analysed with two approaches. Data was first explored to elucidate relationships between larval movement (to or away) to each light treatment and for both early and later released larvae from all species. Ordinations were produced following correspondence analysis based on a chi-squared distance measure. A second approach focused on species-specific responses to light. Here, larval responses to light were coded into a binary choice of attraction or avoidance. The binary data were analysed using a chi-squared goodness of fit test assuming equal attraction and avoidance responses. Responses to each light source, for each species and larval age, were analysed separately.

### Settlement

Laboratory-based larval assays were undertaken to quantify the effect of light on larval settlement. Assays were undertaken in 35-mm PVC Petri dishes. Each Petri dish provided an equal choice of exposure to light or dark by covering one-half of the exterior of the Petri dish (including lid) with black tape. Replication was considered at the Petri dish level with replicates between species differing due to larval supply (n = 25, *I*. *microconnulosa*, *Haliclona* sp., and *L*. *variabilis*., n = 22 *C*. *mathewsi*) while larvae comprised subsamples within replicates (n = 10: *I*. *microconnulosa*, *L*. *variabilis and C*. *mathewsi;* and n = 2 *Haliclona* sp.). Petri dishes were filled with 10 ml of 20 μm filtered seawater (FSW) and larvae were subsequently transferred via a pipette. Petri dish lids were then added ensuring that the covered lid aligned with the covered dish. Settlement assays were undertaken in temperature-controlled rooms at 28°C (equivalent to ambient sweater temperature) and under photoperiod regimes of 12:12. Time to larval settlement in brooded GBR species can occur over one to four days [4, 16, 18, 36, 40, 50, 52, 56] and this guided a decision to score settlement after 3 days. Settlement was defined as metamorphosis where a larva underwent distinct morphological changes that resembled a flattened, disk-like, morphology on the surface substrate. Larval settlement data comprised a binary score of settlement to dark or light sides of each Petri dish. Settlement that occurred on the border of light and dark treatment and metamorphosis at the surface-water interface were also recorded. The distribution of percentage larval settlement between dark and light choices within Petri dishes was analysed using Mann-Whitney U tests.

### Larval fluorescence

Fluorescent microscopy was used to qualitatively assess the presence (or absence) of fluorescent proteins associated with posterior cilia. Larvae were collected (n = 30 per species) and observed within one hour of release. To reduce mobility, and aid imagery, larvae were added to 35-mm Petri dishes containing 5 ml of FSW with the addition of 250 μl of 0.01% Formaldehyde. The location of fluorescence on larvae and the number of larvae exhibiting fluorescence were assessed under a stereo microscope coupled with a Nightsea lamp and fluorescence wavelength Royal Blue filter (excitation 440–460 nm; emission, long pass filter 500 nm).

## Results

### Depth distributions

**Fig 1:**
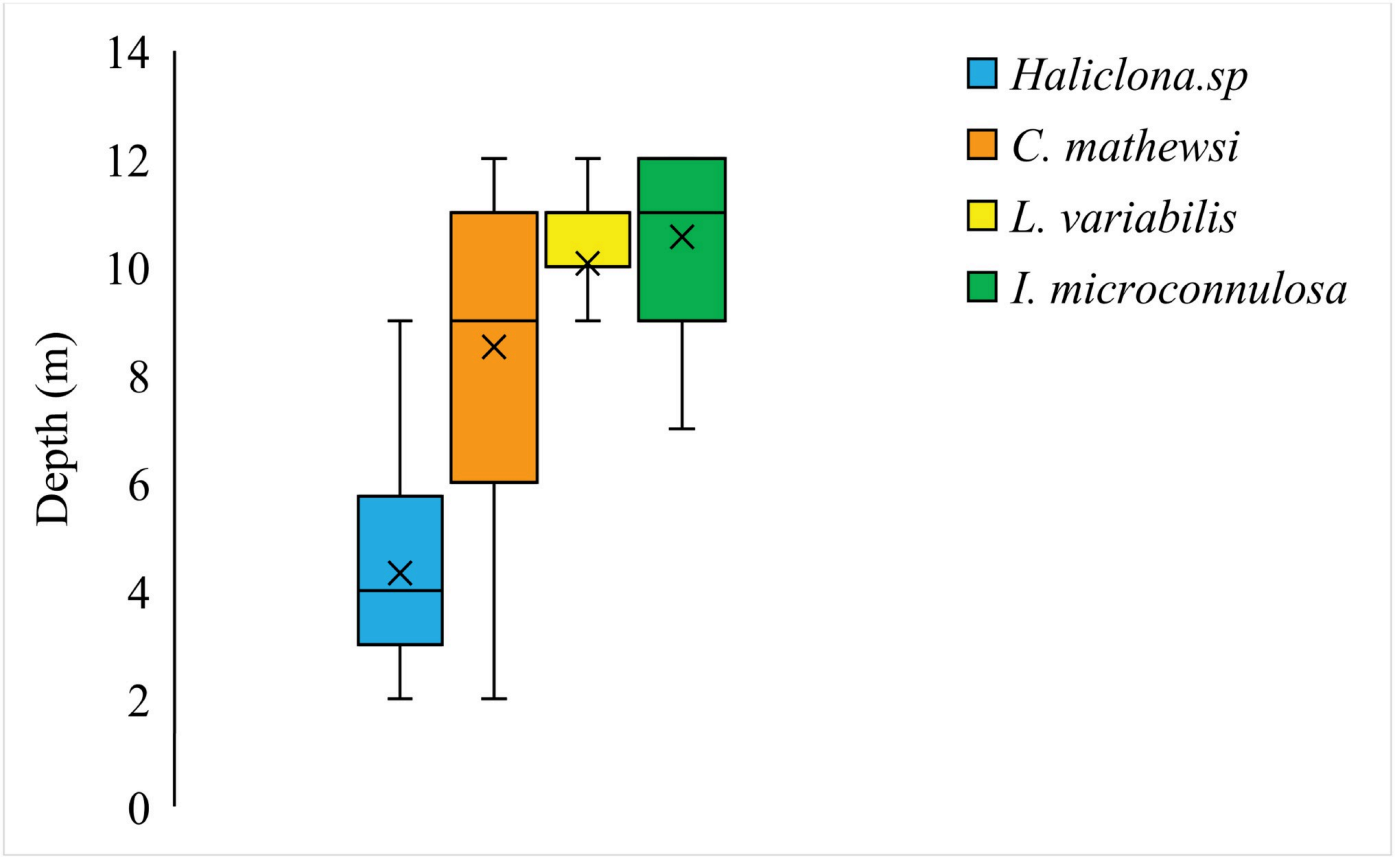
Depth profiles for *Haliclona* sp. (H), C*oscinoderma mathewsi* (CM), *Ircinia microconnulosa* (IM), and *Luffariella variabilis* (LV). Boxplots for each species show data-spread as upper and lower quartiles, median and mean depths (x).

The median depth for *Haliclona* sp. was 4 m compared to 9 m for *C*. *mathewsi* and 11 m for both *L*. *variabilis* and *I*. *microconnulosa* (Fig 1). *Haliclona* sp. *L*. *variabilis* and *C*. *mathewsi* did occur over shallow to deep ranges (*Haliclona*. sp. 2–10 m; *L variabilis* 2–12 m and *C*. *mathewsi* 3–12 m) but no individuals of *I*. *microconnulosa* were observed below 7 m. Depth was a significant factor in species distributions (Welch’s test, F = 163.10_(*3*, *198*.*80*),_ p < 0.01). *Haliclona* sp. occurred in shallower depths (mean 4.32 ± 0.25 m) than the other three species (Tukey’s post hoc test, p < 0.01, all pairwise comparisons), a result also consistent with *C*. *mathewsi* which occurred over significantly different depths to all other species (mean 8.51 ± 0.16 m, p < 0.01 all pairwise comparisons). Both *I*. *microconnulosa* (mean 10.55 ± 0.17 m) and *L*. *variabilis* (mean 10.06±0.16 m) occurred over consistent depths (p = 0.52, Tukey’s pairwise comparison).

### Dispersal

**Fig 2:**
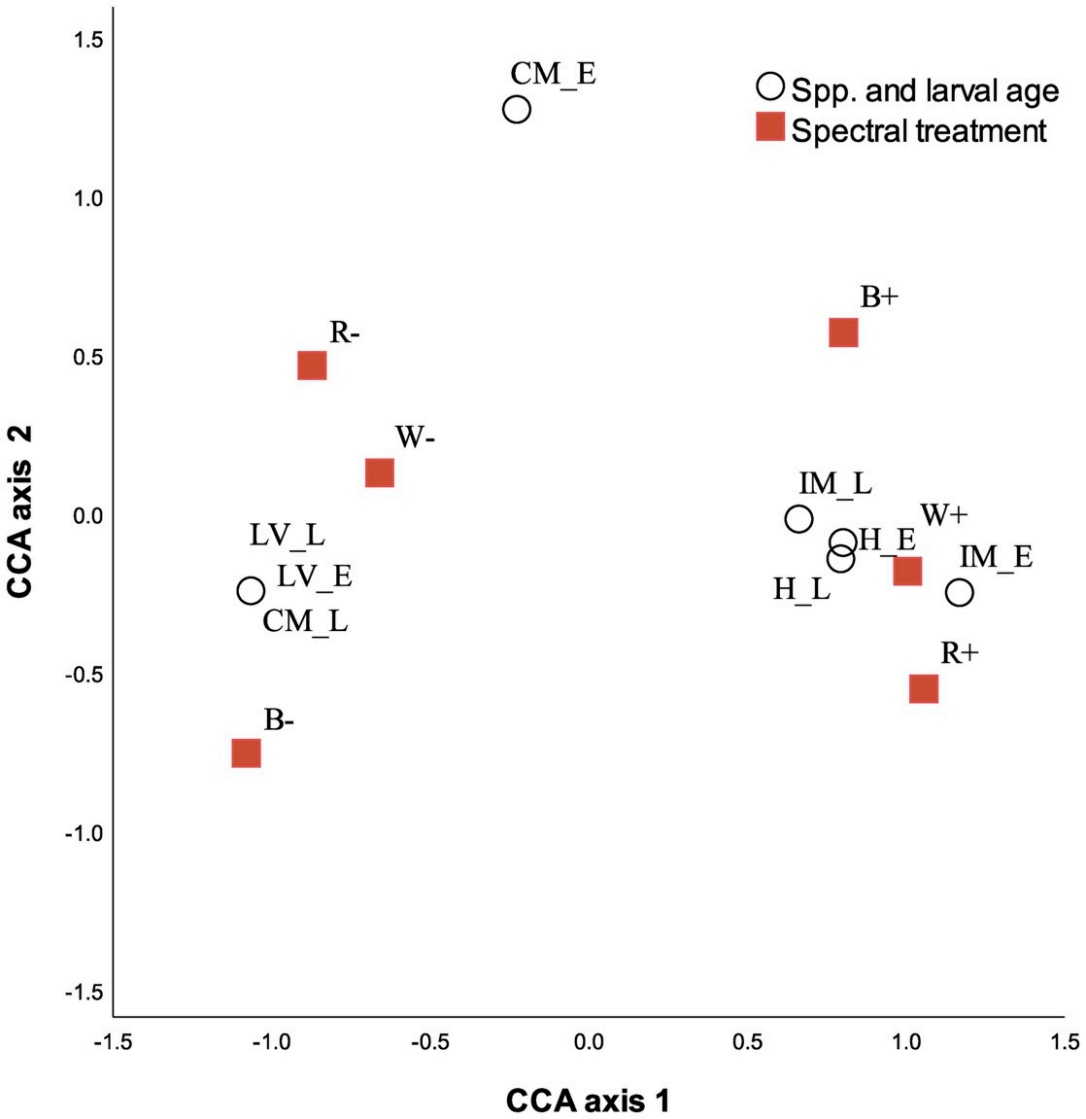
Larval dispersal responses to light for *Ircinia microconnulosa* (IM), C*oscinoderma mathewsi* (CM), *Luffariella variabilis* (LV), and *Haliclona* sp. (H). The ordination, derived from correspondence analysis depicts larval behaviours when exposed to white (W), red (R) and blue (B) light for early (E) and late (L) released larvae. The plot explicitly shows dispersal as measured by attraction (+) to a light source (positive phototaxis) or a photonegative behaviour of dispersing away from a light source (-).

There were interspecific responses of larval dispersal to light and also larval age. An initial exploration of data, based on Correspondence Analysis (CA), showed broad-scale patterns of responses of dispersal to light and also responses moving away from light (Fig 2). This pattern is shown in the ordination where movement to white, red and blue light is positioned on the left of the ordination and clearly separated to the responses avoiding light from all light spectral treatments tested. Both early and late stages of *L*. *variabilis* larvae are separated in the pattern for movement away from light, regardless of the light treatment offered. Conversely, *Haliclona* sp., across both larval ages, drive patterns that separate movement to light, in particular red and white light. Movement patterns for aged *I*. *microconnulosa* larvae, are less clear in the ordination, although there is a weak separation of early to late larvae, with the grouping of both ages of larvae being associated with movement to light. The association of *C*. *mathewsi* early-stage larvae, located in the central-upper part of the ordination, most closely pivots toward the variables of avoidance of red and white light (CA 1) and the attraction to blue light (CA 2).

**Fig 3:**
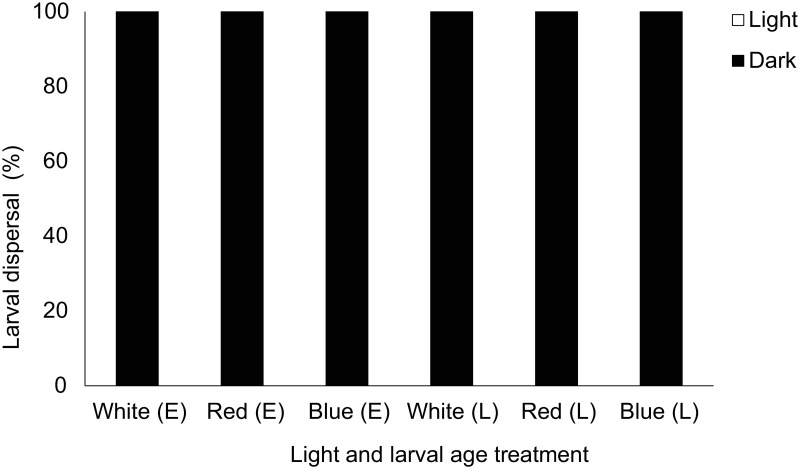
Dispersal responses of *Luffariella variabilis* larvae to light. Each bar represents the percentage of larvae that chose to move towards (Light), or away from (Dark), the light source. Light treatments included white, red and blue light for early (E) and late (L) larvae.

**Fig 4:**
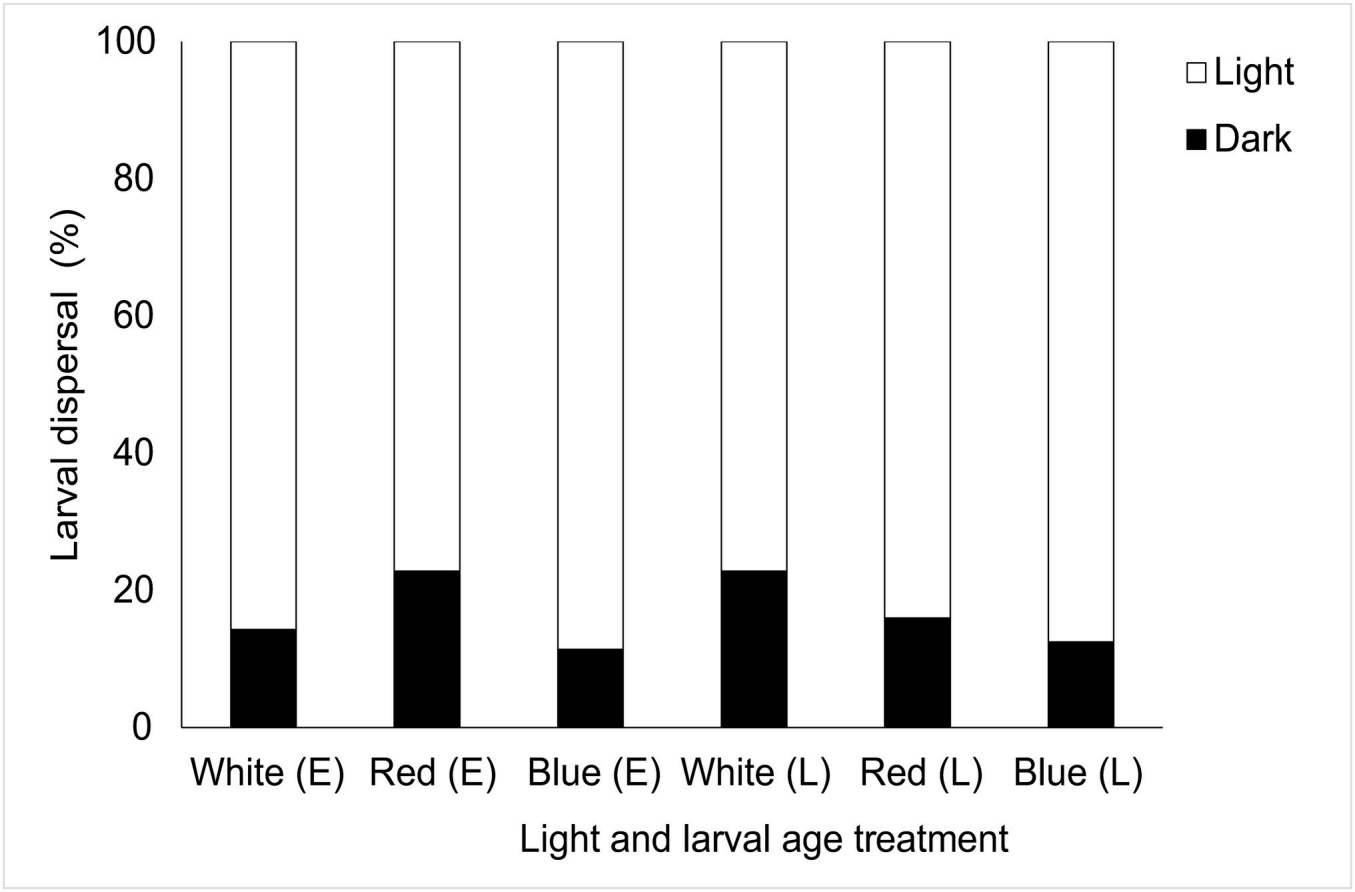
Dispersal responses of *Haliclona* sp. larvae to light. Each bar represents the percentage of larvae that chose to move towards (Light), or away from (Dark), the light source. Light treatments included white, red and blue light for early (E) and late (L) larvae.

**Fig 5:**
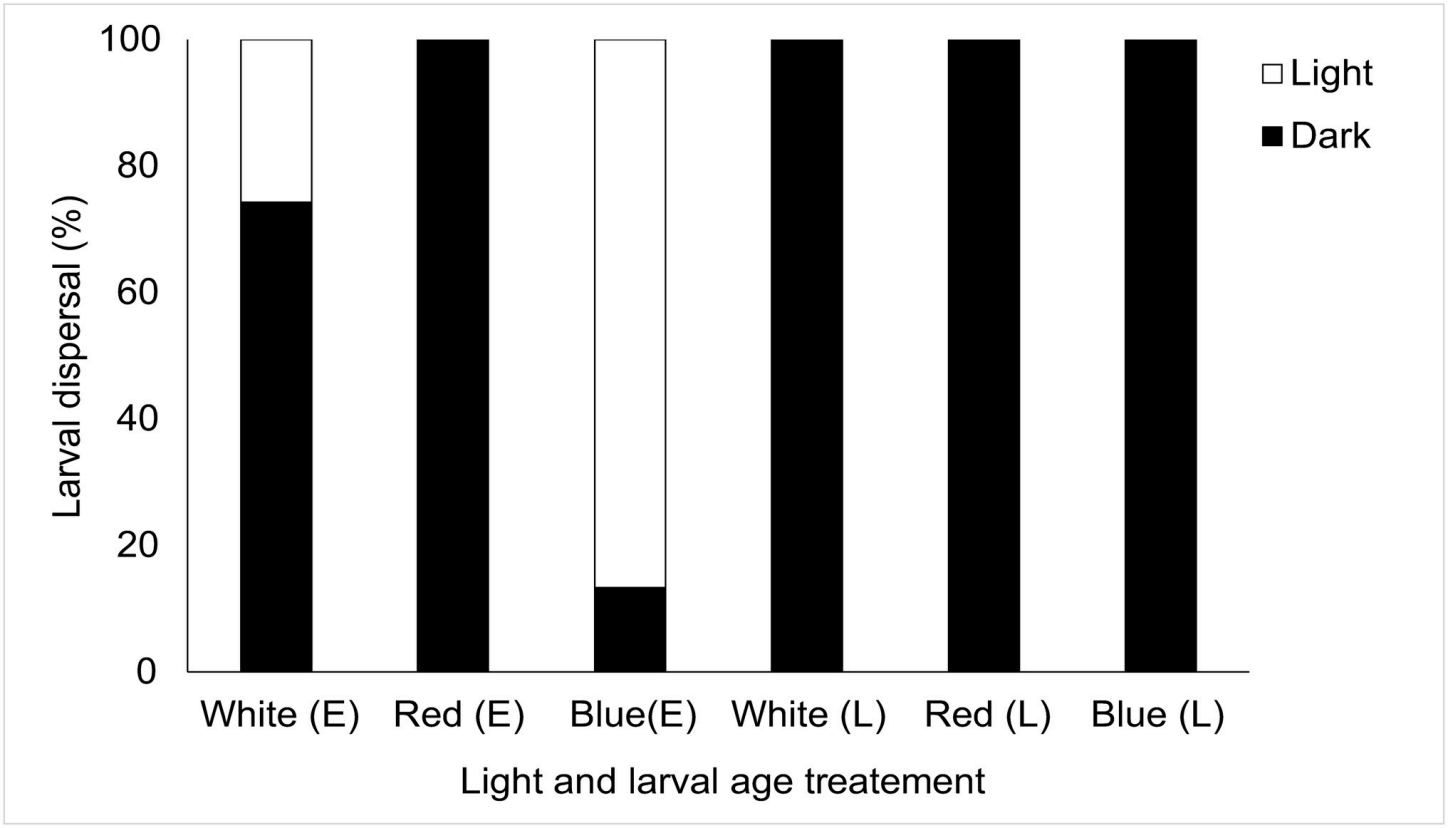
Dispersal responses of *Coscinoderma mathewsi* larvae to light. Each bar represents the percentage of larvae that chose to move towards (Light), or away from (Dark), the light source. Light treatments included white, red and blue light for early (E) and late (L) larvae.

**Fig 6:**
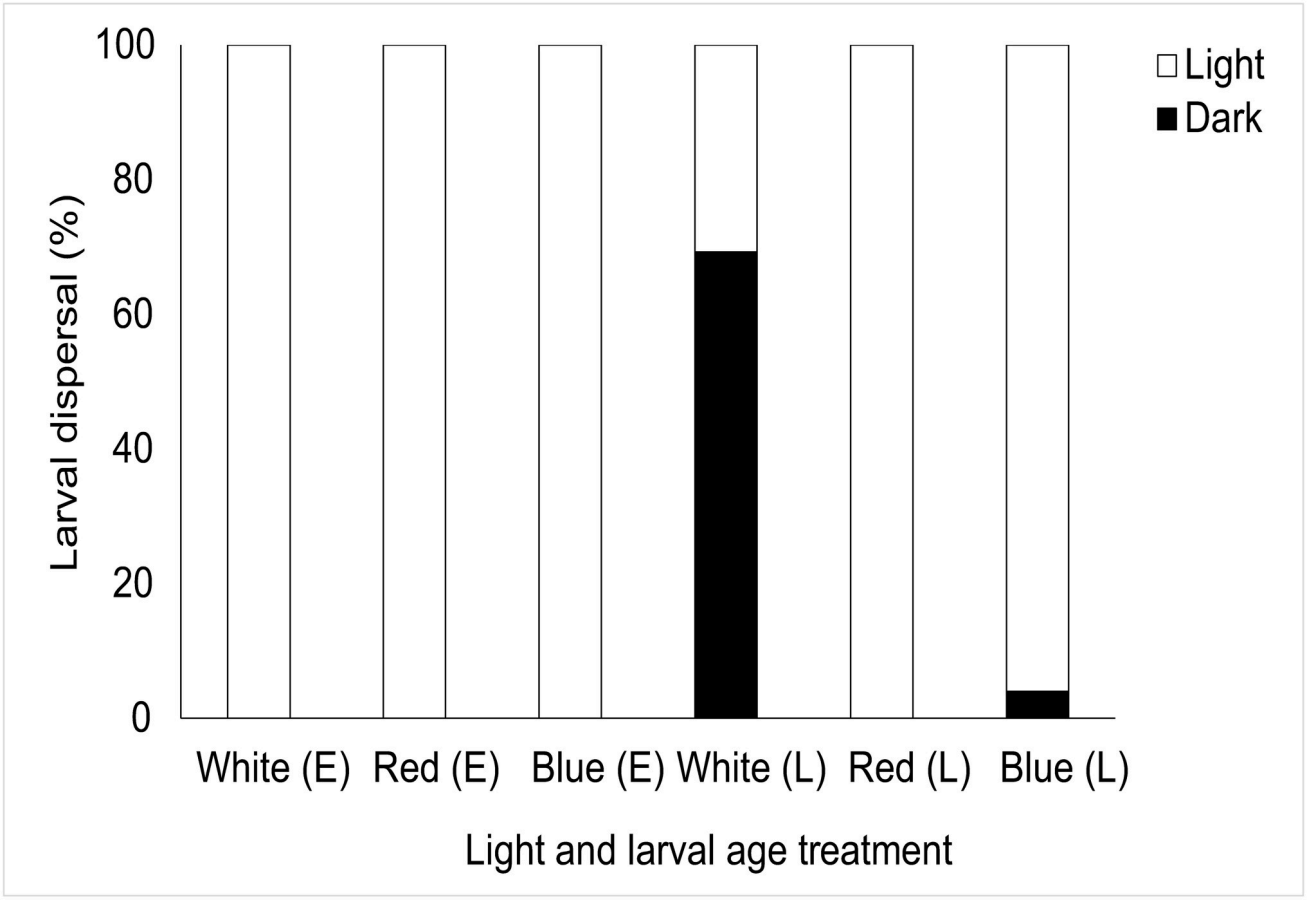
Dispersal responses of *Ircinia microconnulosa* larvae to light. Each bar represents the percentage of larvae that chose to move towards (Light), or away from (Dark), the light source. Light treatments included white, red and blue light for early (E) and late (L) larvae.

More refined analysis showed a significant effect of light on larval behaviour following chi-squared goodness of fit tests across age and light treatment tested (Table 1). Here, *Luffariella variabilis* showed consistent responses moving away from white, red, and blue light in 100% of cases regardless of larval age (Fig 3). Although *Haliclona* sp. showed small percentages of larvae moving away from light (11.43–22.86%) there was a significant overall response to light where larvae moved towards light regardless of light treatment or larval age (Fig 4, Table 1). All (100%) *Coscinoderma mathewsi* late-stage larvae avoided light (all spectral treatments) and there was a significant effect of light on early-stage *C*. *mathewsi* larvae which showed consistent photophobic responses to red (100%) and white (74.29%) light but positively phototactic behaviours to blue light (86.67%) (Fig 5, Table 1). For *I*. *microconnulosa* there were also opposing trends between larval ages and responses to light. Early-stage larvae moved to light in 100% of cases. In older larvae, movement to red light occurred in 100% of cases. There was also a significant movement of older larvae towards blue light (96%) and dispersal away from the white (69.33%) light treatment (Fig 6, Table 1).

**Table 1:** Summary statistics of chi-squared goodness of fit tests for the response of larvae attracted or avoiding white, red and blue light treatments. (a) White, (b) Red, (C) Blue.

Species	χ^2^	DF	Sig	Dispersal response
(a)				
*C*. *mathewsi* early	16.13	1	< 0.001	Away
*I*. *microconnulosa* late	14.44	1	< 0.01	Away
*Haliclona* sp. early	51.84	1	< 0.001	To
*Halcion* sp. late	29.16	1	< 0.001	To
(b)				
*Haliclona* sp. early	29.16	1	< 0.001	To
*Haliclona* sp. late	46.24	1	< 0.001	To
(c)				
*C*. *mathewsi* early	16.13	1	< 0.001	To
*I*. *microconnulosa* late	84.64	1	< 0.001	To
*Haliclona* sp. early	60.84	1	< 0.001	To
*Haliclona* sp. late	57.76	1	< 0.01	To

*L*. *variabilis* (early and late stages) *C*. *mathewsi* (late stages), and *I*. *microconnulosa* (early) resulted in 100% of responses to white light treatments and are not included in a chi-squared goodness of fit test

*L*. *variabilis*, *C*. *mathewsi* and *I*. *microconnulosa* resulted in 100% of responses to light treatments across light spectra and larval age and are not included in a chi-squared goodness of fit test

*L*. *variabilis*, (early and late), *C*. *mathewsi* (late*) and I*. *microconnulosa* (early) resulted in 100% of responses to light treatments across light spectra and larval age and are not included in chi-squared goodness of fit tests

### Settlement

**Fig 7:**
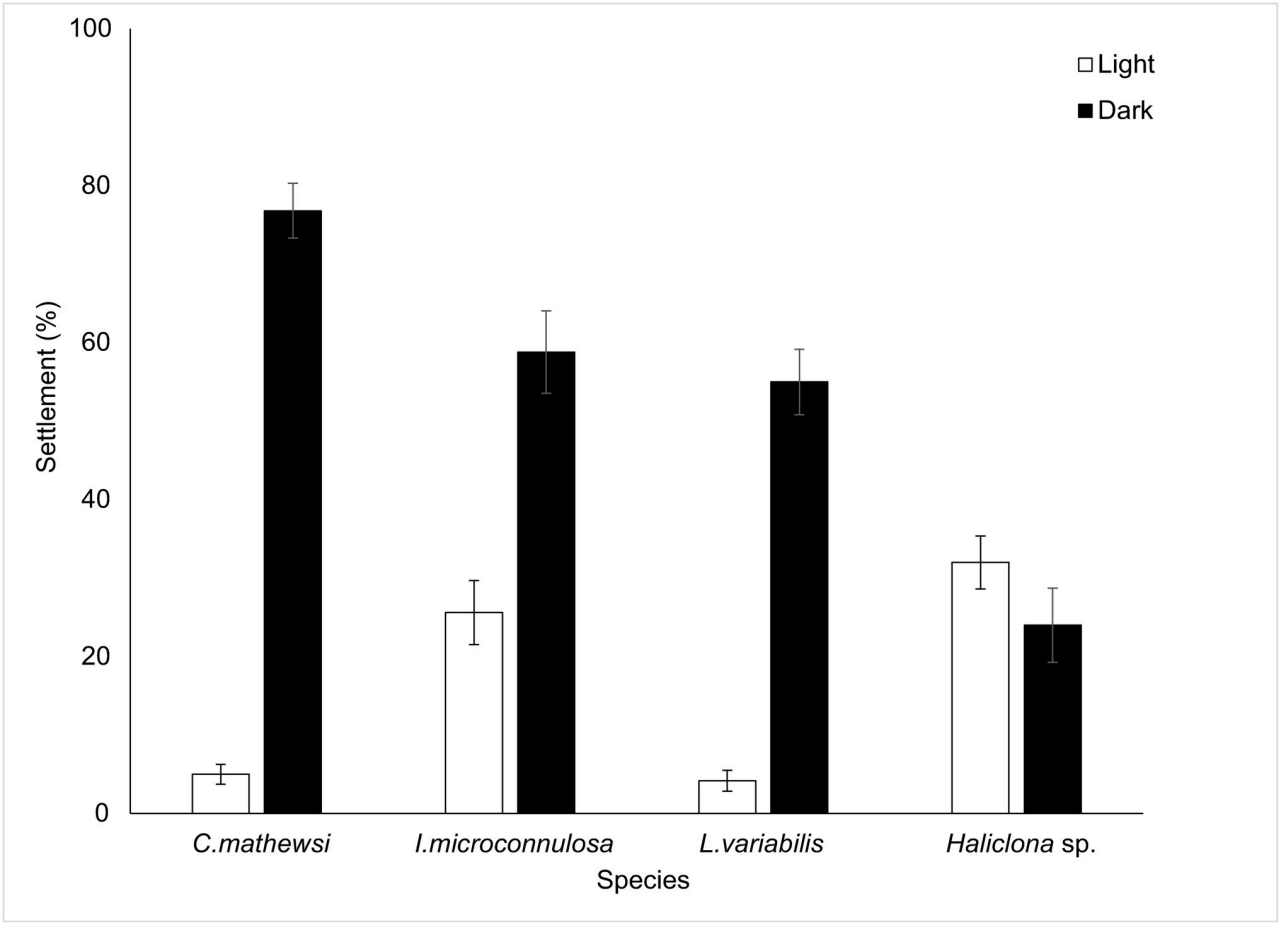
Larval settlement to experimental light treatments. Bars represent mean percentage settlement (±1SE) to light or dark halves of a Petri dish for *Ircinia microconnulosa*, C*oscinoderma mathewsi*, *Luffariella variabilis*, and *Haliclona* sp.

Successful settlement across species ranged from 56% to 83.56% and the role of light on settlement varied among species (Fig 7). There was a significant effect of light on larval settlement, determined by Mann-Whitney U Tests, for *C*. *mathewsi* (U = 484, p < 0.001), *L*. *variabilis* (U = 575, p < 0.001) and *I*. *microconnulosa* (U = 522.50, p < 0.001). Here, settlement to dark was the preferred choice reflected with mean (± 1SE) settlement outcomes of 76.82 ± 3.51%, 58.80 ± 5.27% and 55.00 ± 4.17% for *C*. *mathewsi*, *I*. *microconnulosa* and *L*. *variabilis* respectively. There were consistent levels of mean settlement to both dark (24.00 ± 3.27%) and light (32.00 ± 3.79%) for *Haliclona* sp. (U = 256.5, p = 0.23).

### Larval fluorescence

All four species produced parenchymella larvae which exhibited a prolate spheroid-shape, distinct posterior ciliated tuft and conspicuous darkened bands adjacent to the ciliated tuft (posterior ring). All species had a white body colour, except for *Haliclona* sp. which was pale blue. All larvae (n = 30) from *C*. *mathewsi*, *L*. *variabilis* and *I*. *microconnulosa* showed red fluorescence, visible as a narrow band at the posterior immediately adjacent to the ciliated tuft (S2–S4 Figs). Eighteen of the thirty sampled larvae of *Haliclona* sp. showed red fluorescence bands adjacent to the posterior tuft consistent with *I*. *microconnulosa*, *L*. *variabilis* and *C mathewsi*.

## Discussion

There is a role of light in directing sponge larval dispersal confirming phototactic behaviour for the four species targeted in the present study. Phototactic behaviours were variable among the four species. Two of the targeted species, *Haliclona* sp. and *L*. *variabilis*, showed consistent photobehaviours irrespective of light treatment or larval age treatments. Here, larvae from the shallower-water species, *Haliclona* sp., exhibited positive phototaxis while larvae from the deeper-water sponge, *L*. *variabilis*, were negatively phototactic. The findings for both *Haliclona* sp. and *L*. *variabilis* support other studies where sponge larvae show consistent negative or positive phototactic behaviours throughout their larval phase [18, 38, 52–55].

*Coscinoderma mathewsi* and *I*. *microconnulosa* exhibited dynamic phototactic behaviours, particularly with larval age. *Coscinoderma mathewsi* larvae are positively phototactic at release (blue light) but are negatively phototactic to all other light treatments, irrespective of age. *I*. *microconnulosa* switch from positive to negative phototaxis as larvae age (white light). Other studies document temporal phototactic behavioural responses for sponge larvae [39], interpreted as being a contributor to larval dispersal (40), but these studies focus on experiments using white light. Brooded sponge larvae are commonly released during daylight hours and can be planktonic for hours to days [18, 40, 52, 56–60]. Sponge larvae are weak swimmers and unlikely to disperse effectively when exposed to hydrodynamics [61] but the use of light to guide navigation may assist dispersal via vertical migration, as is suggested for several invertebrate larvae, including sponges [40, 62]. Sponges that spawn during the day would facilitate photobehaviours in larvae, contributing to upward movement into the water column for positive phototactic larvae, and to the benthos or cryptic habitats, for negative phototactic species [18, 40]. Most sponge larvae reported to date are planktonic for hours to days [61], and therefore exposed to variable diurnal/nocturnal cycles so moonlight may also play a role in light-driven dispersal behaviour; there are no studies directly documenting behaviours to moonlight and there would be value in examining the role of diurnal cycles and the source of celestial light (sun or moon) on phototaxis for dispersing sponge larvae.

Our knowledge of sponge larval photobehaviours is presently informed by studies that have relied on white light sources (sunlight or experimental sources). Leys et al. [37] provided data on *Amphimedon queenslandica* (cilia) responses to light spectral components but there appears to be nothing on (whole) sponge larval dispersal behaviours to selected wavelengths within white light that attenuate with depth. In the present study, deeper-water species, *L*. *variabilis*, exhibited a negative phototactic behaviour confirming previous studies using white light sources [18]; this species also avoided the red and blue light experimental treatments suggesting a choice to disperse toward light-limited habitats associated with depth or cryptic habitats, a result confirmed with the depth distribution of this species. Conversely, *Haliclona* sp. showed positive phototactic behaviour irrespective of wavelength treatment, and this behaviour would contribute to dispersal to illuminated habitats, associated with shallow water, also observed with the predominant depth distributions of these sponges. That *L*. *variabilis* and *Haliclona* sp. showed consistent phototactic behaviours, irrespective of light treatment, suggests photobehaviours of these species are actioned to any light wavelength.

Of interest, is the behavioural response to the spectral treatments of light for *C*. *mathewsi* and *I*. *microconnulosa*, which both showed a distinction to detect and respond to spectral wavelengths. Aged *I*. *microconnulosa* had a negative phototactic behavioural response to white light but responded to blue and red light by dispersing toward both light spectra. Early stage *C*. *mathewsi* also dispersed to blue light. Sessile marine invertebrate larvae that show a behavioural response to light spectra, while not reported in sponges, are reported in other taxonomic groups. For example, some coral larvae appear to have spectral sensitivity to blue light in particular, assisting dispersal to deeper habitats where longer wavelengths of light (e.g. red) attenuate with depth [63]. Other coral species exhibit spectral sensitivity to red light to assist with depth/habitat dispersal [31]. Findings of larval photobehaviours from other taxa (e.g. corals), coupled with the results on blue light responses of larvae in the present study and in cilia by Leys et al. [37], provide plausibility that some sponge larvae can detect particular wavelengths to assist dispersal contributing to settlement to favourable habitats and therefore adult survival [20, 32].

A second question of this study asked what role light played in larval settlement, and whether photobehaviours in the dispersal phase correspond to settlement patterns. While there was no effect of light in the settlement of the shallower-water species, *Haliclona* sp., the three deeper-water species showed a preference to settle to shaded treatments, supporting a premise that these species seek habitats that are associated with light attenuated depths. Settlement to shaded treatments can also include cryptic habitats in both deep and shallow water and this scenario cannot be discounted, as evidenced by sponge larval settlement to microtopography to either avoid light or optimize light exposure [16, 64]. *Coscinoderma mathewsi*, a deeper-water species settled to shaded treatments, was negatively phototactic to white and red light but was positively phototactic to blue light (early-stage larvae) supporting behaviour that would contribute to dispersal and settlement to deeper habitats. *I*. *microconnulosa* showed contrasting behavioural patterns to other deeper-water species. Early-stage larvae of this species are positively phototactic, which would support an interpretation of larvae initially moving upward into the water column. With age, larvae avoid white light (negatively phototactic) and showed positive phototaxis to blue and red light. The avoidance of white light, and movement to blue light in aged larvae would support an interpretation of dispersal to deeper habitats, but this interpretation is tempered with the result that aged larvae also move towards red light. Nevertheless, the photobehaviours of both *I*. *microconnulosa* and *C*. *mathewsi* highlight the capacity of larvae to sense, process and respond to the environmental signals within a light spectrum to support ecologically relevant decisions for dispersal and settlement.

Photoreceptive diversity is exemplified in single-celled organisms to eyed vertebrates, contributing to photo-driven-movement in both prokaryotes and eukaryotes [22]. A reliance on photoreception across life is not surprising given the detection and reactions to light support critical life processes including photosynthesis, the ability to synchronize with environmental cycles (circadian clocks), and to mitigate damage associated with ultraviolet radiation [22, 30]. Sponges represent a group without obvious visual detection systems or eyes. The use of light-sensitive cells, coupled to a ciliated posterior, orientate and propel larvae [38, 39] and has been suggested as a functional eye in this group [34]. In other animals, including corals [65, 66]), the conversion of photons of light to electrical signals occur with the use of photosensitive proteins (opsins), but these protein families are yet to be detected in sponges. Leys et al. [37, 38] demonstrated larvae of *Amphimedon queenslandica* are phototactic, exhibit posteriorly located photosensitive cells, and proposed that light detection was associated with flavins, carotenoids or cryptochromes. Following Leys et al., [37] Rivera et al. [34] further established that cryptochrome genes occur in *A*. *queenslandica* and likely contribute to larval photobehaviour. Cryptochromes are a recognized light detection system in other taxonomic groups [67, 68], and also represent a plausible pathway for light sensing in sponge larvae. Nevertheless, *A*. *queenslandica* is the sole species informing this proposal for sponges and highlights the value of considering other light-detecting pathways and species. Discrete red fluorescent rings were observed in all sampled larvae of *L*. *variabilis*, *C*. *mathewsi* and *I*. *microconnulosa*. In *Haliclona* sp., fluorescence was not detected in 40% of larvae and further work is required to resolve why fluorescence was not observed. It is unlikely that this result is due to larvae being spawned from different species given the conspicuous adult (and larval) morphology of *Haliclona* sp. A more plausible explanation would be the intensity of fluorescence was diminished/extinguished with the use of formaldehyde thereby impeding detection in this species; the formaldehyde slowed larval movement to observe and photograph individuals but also resulted in mortality within several minutes. For the larvae that did show fluorescence in this study their proximal location to posterior cellular rings raises the question of whether these fluorescent bands are proteins that also contribute to light detection, potentially in tangent with cryptochromes. Fluorescent proteins have been implicated in light-driven electron transfer, suggesting a functional role in light detection in a cnidarian [45] however, the overall photobiological role of fluorescent proteins is still largely unresolved [32]. While further work is required to inform sensible arguments that fluorescent proteins contribute to light detection in sponge larvae, the clear concentrated band of fluorescence at the region of light-photosensitive cells and posterior ciliated tuft, coupled with larval photobehaviours, highlights their potential as a line of further investigation for light sensing in this group.

## Conclusions

Behavioural response to light includes the use of positive and negative phototaxis. Under experimental conditions, this study identified the interspecific responses of sponge larvae to light highlighting the potential for some species to detect and respond to discrete wavelengths of light. While recognizing the complexity of light in natural aquatic systems, and the limitation of targeting responses to monochromatic light in laboratory-based settings, the findings from this experimental study highlight that aneural sponge larvae have the ability to disperse to light-conditioned habitats. Without the opsin-regulated light detection systems seen in other animals, the use of cryptochromes to detect light, as proposed for *A*. *queenslandica*, are likely to be important for other sponges; the use of fluorescent proteins, evidenced as discrete fluorescent bands in this study, may also contribute to how sponge larvae sense light, ultimately contributing to directed dispersal and settlement to habitat.

## Supporting information

S1 Fig Experimental light chamber. Chamber comprises vertical PVC pipe uprights and a horizontal connector pipe. The light source, secured to a retort stand, is orientated into a clear acrylic window of the vertical upright pipe to provide a gradient of light along the horizontal connector pipe.
(TIF)

S2 Fig Fluorescence in a *Ircinia microconnulosa* larva. Photomicrograph depicts red fluorescent band (F) contiguous to larval posterior ring (PR) and ciliated tuft.
(TIF)

S3 Fig Fluorescence in a *Haliclona* sp. larva. Photomicrograph depicts red fluorescent band (F) contiguous to larval posterior ring (PR) and ciliated tuft.
(TIF)

S4 Fig Fluorescence in *Luffariella variabilis* larvae. Photomicrograph depicts red fluorescent band (F) contiguous to larval posterior ring (PR) and ciliated tuft.
(TIF)

S5 Fig Fluorescence in *Coscinoderma mathewsi* larvae. Photomicrograph depicts red fluorescent band (F) contiguous to larval posterior ring (PR) and ciliated tuft.
(TIF)

S1 Table Sample sizes for early and late stage larval trials. (PDF)
