# Supplementary figures and images for: The role of photobehaviour in sponge larval dispersal and settlement
Source: PLoS One. 2023 Jul 10;18(7):e0287989. doi: 10.1371/journal.pone.0287989 (PMC10332607; doi:10.1371/journal.pone.0287989)

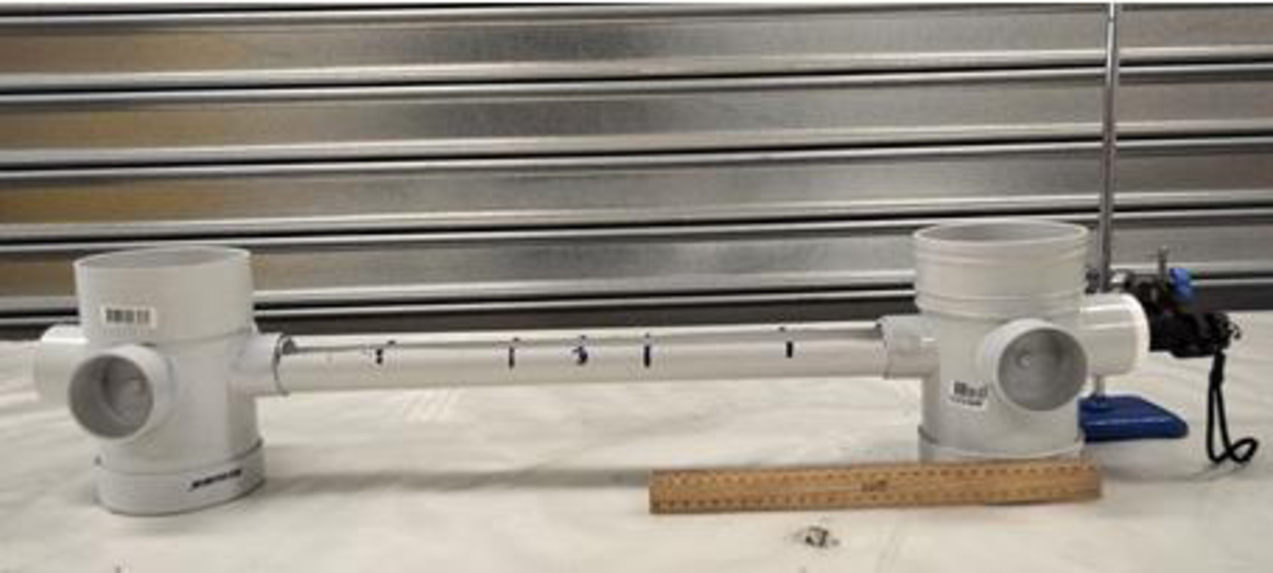

Supplement: S1 Fig — Chamber comprises vertical PVC pipe uprights and a horizontal connector pipe. The light source, secured to a retort stand, is orientated into a clear acrylic window of the vertical upright pipe to provide a gradient of light along the horizontal connector pipe. (TIF) [file pone.0287989.s001.tif]

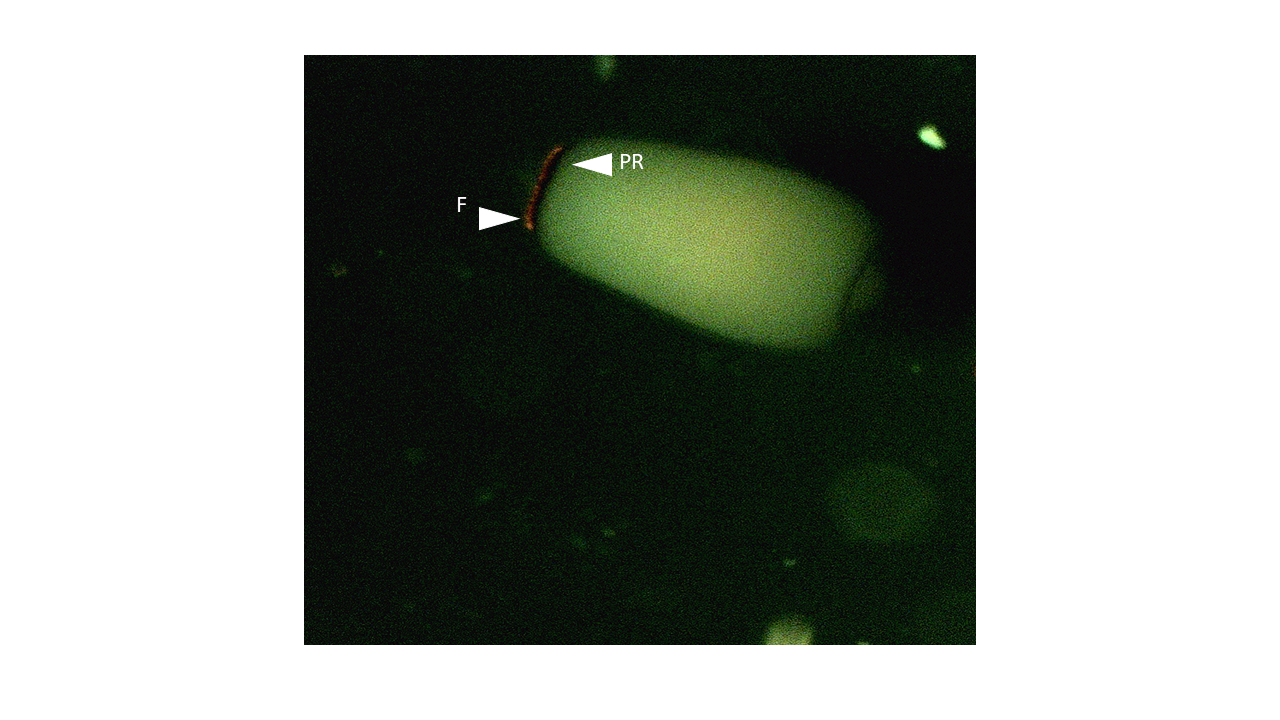

Supplement: S2 Fig — Photomicrograph depicts red fluorescent band (F) contiguous to larval posterior ring (PR) and ciliated tuft. (TIF) [file pone.0287989.s002.tif]

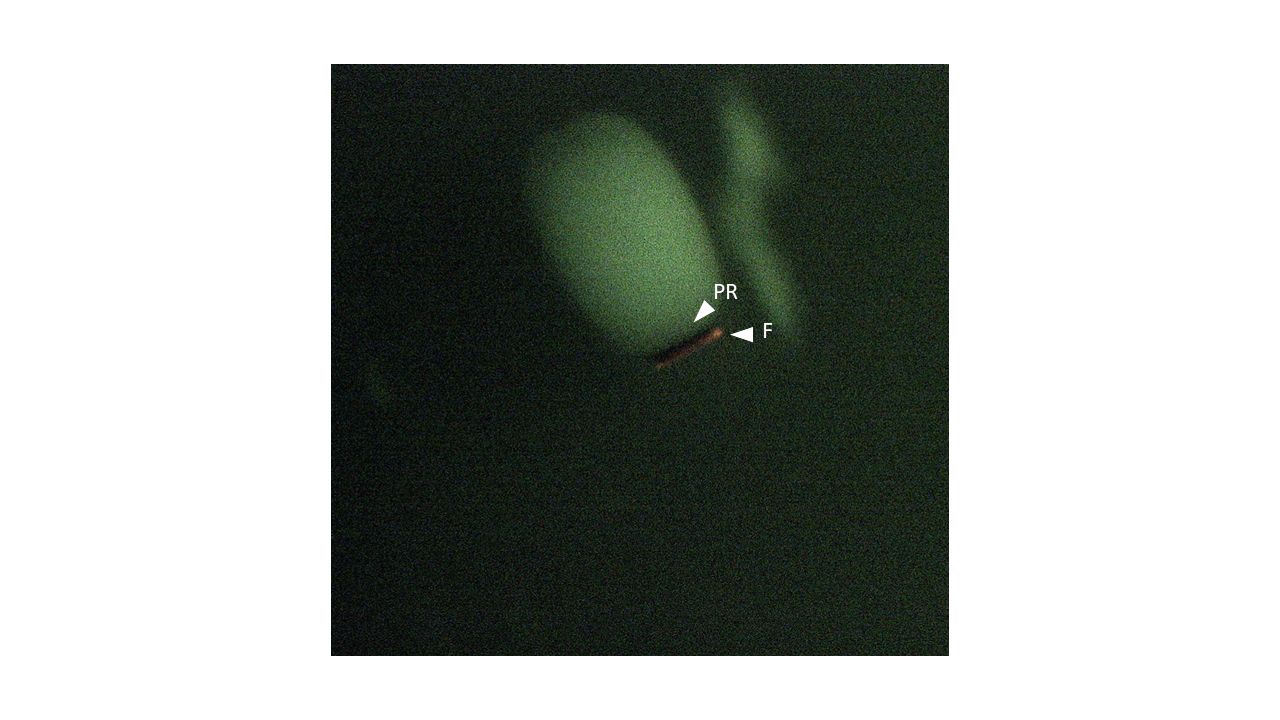

Supplement: S3 Fig — Photomicrograph depicts red fluorescent band (F) contiguous to larval posterior ring (PR) and ciliated tuft. (TIF) [file pone.0287989.s003.tif]

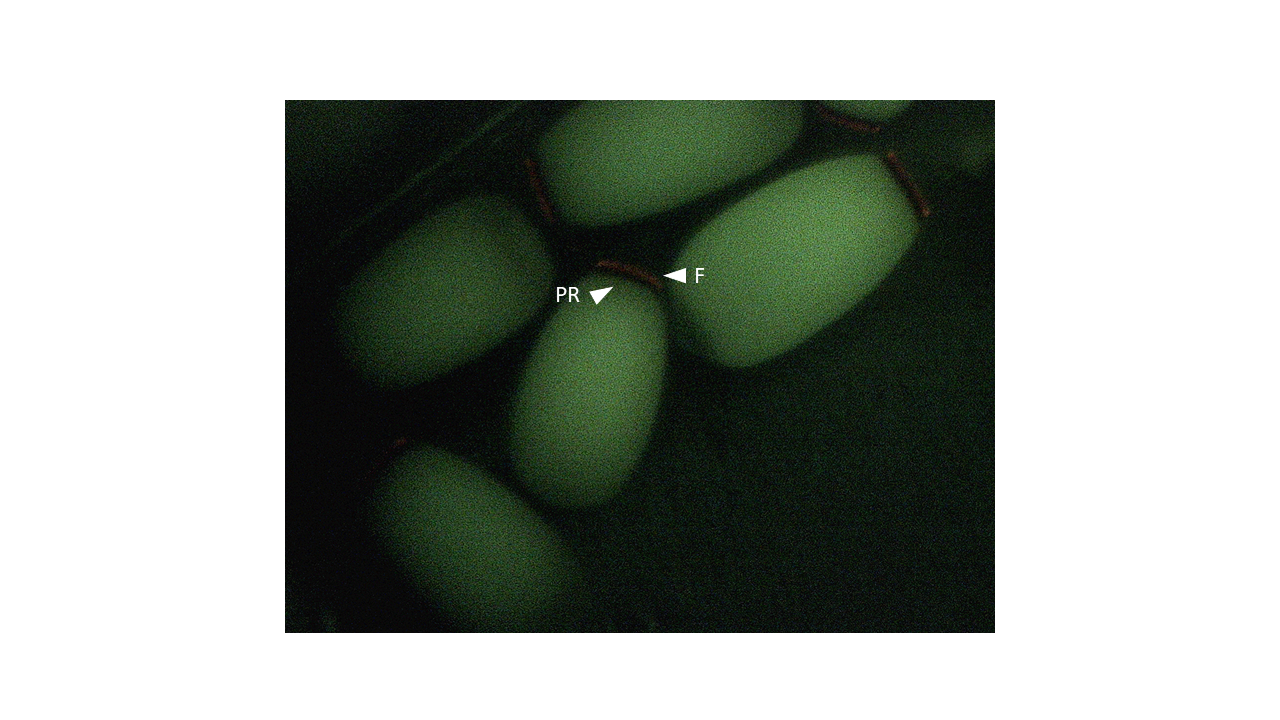

Supplement: S4 Fig — Photomicrograph depicts red fluorescent band (F) contiguous to larval posterior ring (PR) and ciliated tuft. (TIF) [file pone.0287989.s004.tif]

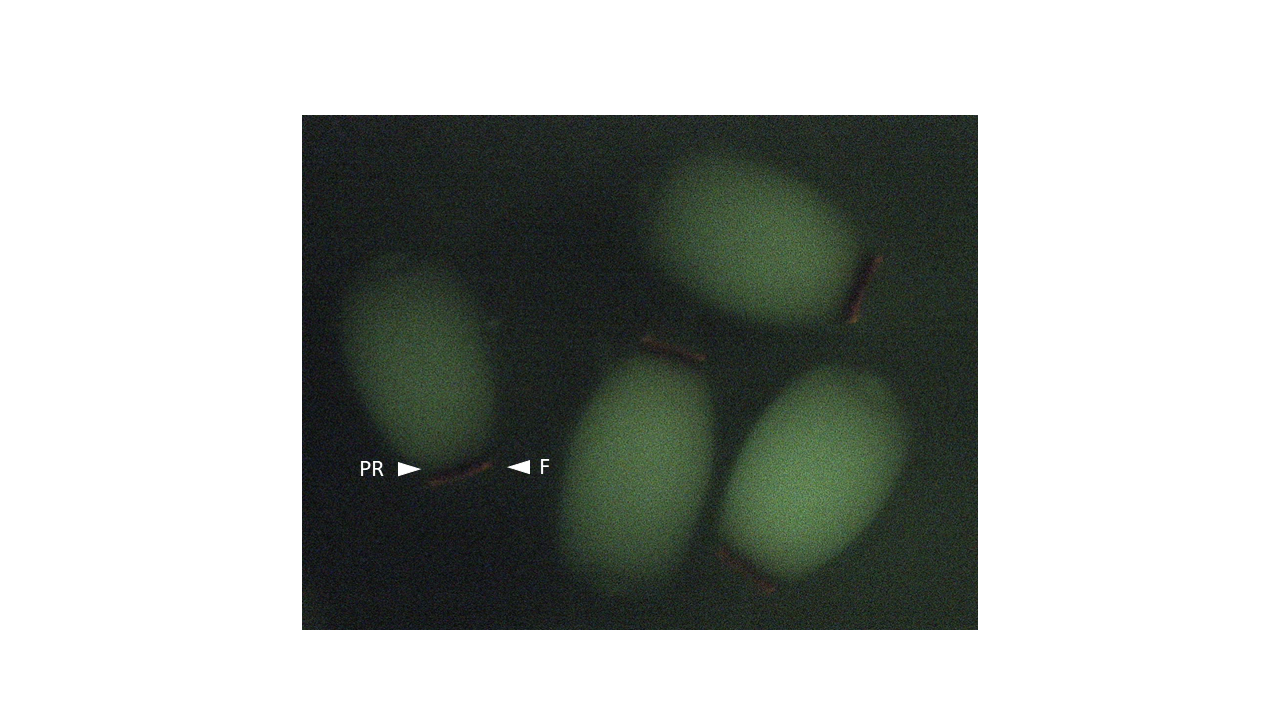

Supplement: S5 Fig — Photomicrograph depicts red fluorescent band (F) contiguous to larval posterior ring (PR) and ciliated tuft. (TIF) [file pone.0287989.s005.tif]
