# Supplementary material for: The role of photobehaviour in sponge larval dispersal and settlement
Source: PLoS One. 2023 Jul 10;18(7):e0287989. doi: 10.1371/journal.pone.0287989 (PMC10332607; doi:10.1371/journal.pone.0287989)
Supplement: S1 Table — (PDF) [file pone.0287989.s006.pdf]

Table S1: Sample sizes for early and late stage larval trials.

	Early			Late		
**Species**	**White**	**Red**	**Blue**	**White**	**Red**	**Blue**
Haliclona sp.	35	35	35	70	75	48
Ircinia microconnulosa	37	40	37	75	75	75
Coscinoderma mathewsi	35	35	30	65	55	70
Luffariella variabilis	35	35	35	75	75	75
